# Recommendations for optimising pilot and feasibility work in surgery

**DOI:** 10.1186/s40814-024-01489-1

**Published:** 2024-04-18

**Authors:** K. Fairhurst, S. Potter, J. M. Blazeby, K. N. L. Avery

**Affiliations:** Centre for Surgical Research, Medical Research Council ConDuCT-II Hub for Trials Methodology Research and Biomedical Research Centre, Department of Population Health Sciences, Bristol Medical School, University of Bristol, Canynge Hall, 39 Whatley Road, Clifton, Bristol BS8 2PS UK

**Keywords:** Surgeons, Feasibility studies, Trials

## Abstract

**Background:**

Surgical trials are recognised as inherently challenging. Pilot and feasibility studies (PAFS) are increasingly acknowledged as a key method to optimise the design and conduct of randomised trials but remain limited in surgery. We used a mixed methods approach to develop recommendations for how surgical PAFS could be optimised.

**Methods:**

The findings from a quantitative analysis of funded surgical PAFS over a 10-year period and in-depth qualitative interviews with surgeons, methodologists and funders were triangulated and synthesised with available methodological guidance on PAFS.

**Results:**

The synthesis informed the development of an explanatory model describing root causes and compounding challenges that contribute to how and why surgical PAFS is not currently optimised. The four root causes identified include issues relating to (i) understanding the full scope of PAFS; (ii) design and conduct of PAFS; (iii) reporting of PAFS; and (iv) lack of appreciation of the value of PAFS by all stakeholder groups. Compounding challenges relate to both cultural issues and access to and interpretation of available methodological PAFS guidance. The study findings and explanatory model were used to inform the development of a practical guidance tool for surgeons and study teams to improve research practice.

**Conclusions:**

Optimisation of PAFS in surgery requires a cultural shift in research practice amongst funders, academic institutions, regulatory bodies and journal editors, as well as amongst surgeons. Our ‘Top Tips’ guidance tool offers an accessible framework for surgeons designing PAFS. Adoption and utilisation of these recommendations will optimise surgical PAFS, facilitating successful and efficient future surgical trials.

**Supplementary Information:**

The online version contains supplementary material available at 10.1186/s40814-024-01489-1.

## Background

Surgical research is fundamentally challenging because surgery is a complex intervention. Unlike in pharmacological studies, surgical interventions are more complicated to standardise, define and compare with other interventions [1]. The challenges of designing, conducting and evaluating surgical trials have been well documented [2–7] and may be considered largely related to methodological or cultural issues. Key methodological challenges of surgical trials include recruitment, intervention stability and standardisation, and outcome selection and/or measurement. Cultural issues often compound methodological challenges and include problems with equipoise amongst surgeons, inexperience recruiting to Randomised Controlled Trials (RCTs) and lack of understanding of the multidisciplinary (non-clinical) expertise needed for definitive trial success. Although many of the practical and methodological challenges of evaluating surgical interventions are common to the assessment of all complex, non-pharmacological interventions, surgery is unique in that multiple challenges often coincide [2].

Pilot and feasibility studies are increasingly recognised as a key component for the success of subsequent definitive trials (studies appropriately powered to achieve an effect size and consequently definitively answer a research question). Definitions of the terms ‘pilot’ and ‘feasibility’ have evolved in the literature over time, with terms often used interchangeably and without universally accepted definitions [8–10]. Several major funders in the UK such as the National Institute for Health and Care Research (NIHR) and the Medical Research Council (MRC), have now adopted the conceptual framework to classify pilot and feasibility studies published by Eldridge et al. in 2016 [8]. However, for the purposes of this work, pilot and feasibility studies (PAFS) are defined broadly as ‘Any research undertaken before a main study that is explicitly intended to inform the design and/or conduct of a future main study.’

PAFS have value in informing the design and conduct of surgical trials because they face unique complexities, and often interacting uncertainties surrounding the design, conduct and completion of trials, meaning there is an even greater need to consider if and how surgical trials can be improved. Indeed, funders encourage consideration of feasibility before an agreement to fund a definitive trial is reached [11]. PAFS may help avoid poor research design, conduct and analysis, all of which are known to contribute to significant research waste [12–16]. By avoiding common problems such as the inability to recruit and a corresponding reduction in statistical power, excessive attrition due to intolerable procedures and cross-over between treatment groups, it has been suggested that PAFS may reduce the proportion of failed trials [17].

Whilst published guidance from the MRC [1, 18] and the IDEAL (Idea, Development, Evaluation, Assessment, Long-term follow-up) collaboration [19, 20] emphasises the importance of PAFS for trials of complex interventions such as surgery, accessible practical guidance tailored specifically to optimally design and undertake PAFS for surgical trials is lacking. The conduct, reporting and publication of PAFS in surgery remain rare [21, 22], and guidance such as the IDEAL recommendations, a framework for evaluating and reporting surgical innovation, has yet to lead to a demonstrable improvement in the performance and publication of surgical PAFS [23]. Furthermore, there is no surgery-specific guidance endorsed by funding bodies or professional membership organisations such as the Royal College of Surgeons (RCS) tailored to surgeons participating in or contemplating designing PAFS.

Accessible guidance specifically for surgeons, which distils the important methodological messages for designing and conducting PAFS into a practical useable framework, is needed. The aim of this work was, to generate a detailed understanding of the challenges of PAFS in surgery and to develop clear and practical recommendations for surgeons for how to optimise the design and conduct of surgical PAFS in the future. Such guidance will improve the understanding of the true purpose of PAFS in the context of surgical research, drive up the quality of research applications, optimise reporting and ultimately improve the quality and value of surgical RCTs, thereby significantly reducing research waste.

## Methods

**Fig. 1:**
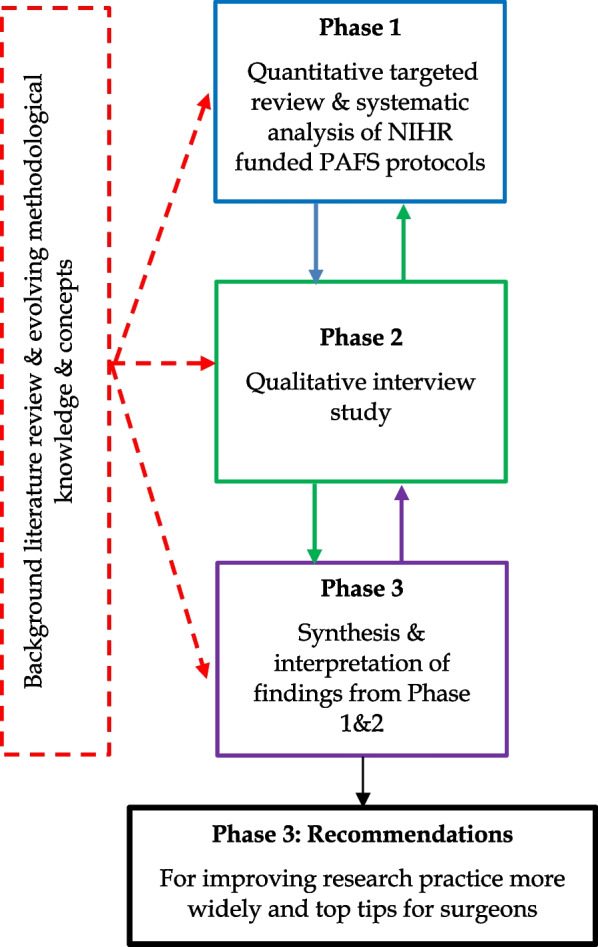
Study process of data collection, synthesis and analysis

Our recently published 10-year review of NIHR-funded surgical PAFS [24] established that the full potential of PAFS to address the uncertainties and challenges specific to undertaking surgical trials is yet to be achieved. Findings from this review and from in-depth qualitative interviews with key stakeholders exploring the challenges and barriers to undertaking PAFS in surgery were synthesised to identify key factors contributing to sub-optimal surgical PAFS and develop practical recommendations for the design and conduct of PAFS in surgery. The synthesis triangulated data from both the quantitative review and the qualitative interviews, with available guidance on the importance of PAFS for trials of complex interventions [1, 19, 20] and wider methodological literature on PAFS more generally [8, 25–29]. Figure 1 illustrates the study process.

### Interview study participant sampling

A list of potential participants was created by considering a long list of trial team members of studies included in our published 10-year review of NIHR-funded surgical PAFS [24]; Senior members of clinical trial units (CTUs) and RCS Trials Centres in the UK; Senior authors of published literature on PAFS work methodology; Senior editors of surgical journals and those publishing PAFS specifically and; Senior funding panel members of major funding bodies in the UK including NIHR, Cancer Research UK (CRUK), Chief Scientist Office (CSO) and Arthritis Research UK (ARUK). Senior participants were deliberately targeted as it was hypothesised they would have the greatest breadth and range of experience in the area of PAFS design and conduct, to consequently allow the extraction of information about the challenges and barriers to actually conducting pre-trial research from those with the most extensive experience. Participants were purposively sampled to achieve maximum variation based on the geographical place of work, clinical vs non-clinical expertise; clinical speciality (if applicable), areas of expertise and research roles. Snowballing sampling was also used, by asking participants during the interviews, if they had suggestions for other participants who may have alternative, relevant or important perspectives for this work.

### Interview data collection and analysis

Interviews were conducted either face-to-face or by telephone at times and locations convenient to the participants, using a topic guide. This semi-structured approach provided a tool to frame the interviews and offer prompts for the interviewer whilst also allowing key topics of importance to participants to emerge naturally and be further explored (Appendix 1). Data analysis used an inductive thematic approach underpinned by the principles of grounded theory [30] using NVivo 10 software [31]. Sampling, data collection and analysis were undertaken concurrently and iteratively until no new themes emerged and data saturation was achieved.

## Results

### Demographics of Interview study participants

A total of 33 participants were invited to participate in an interview of whom 28 (85%) expressed an interest and 27 (81%) consented and were interviewed. These included 18 (67%) males and 9 (33%) females. Of the interviewed participants, 11 (41%) were surgeons, 16 (59%) methodologists and 20 (74%) funders. Surgeon participants included representatives of a broad spectrum of surgical specialties from centres across the UK. All the surgeon participants were currently involved in surgical research, but experience of involvement in PAFS varied widely. Trial methodologist participants included CTU directors and trial statisticians, all of whom reported experience in designing and conducting trials of complex interventions and PAFS, with a smaller proportion having had specific experience in surgical trials (3/16).

More than two-thirds (20/27) of participants interviewed had current or recent experience of membership on a UK research funding body panel including as a panel chair (*n* = 6), deputy chair (*n* = 3) or panel member (*n* = 12). More than half (15/27) were members of journal editorial boards and most (24/27) also currently held a professorial position at a UK university. Table 1 shows further interview participant demographics.

**Table 1:** Interview participant (*n* = 27) summary of demographic characteristics and surgical research/trials experience

Demographic	Number					
Sex	Male			Female		
	18			9		
Interview length	Mean (minutes)			Range (minutes)		
	58			27–101		
Interview mode	Telephone			Face to Face		
	17			10		
Clinical role	Surgeon			Other clinical specialty		
	11 (*n* = 1 previous)			5 (*n* = 4 previous)		
Roles held currently/recently relating to research and/or trials						
Trial involvement	CI	CTU director		Statistician	Methodologist	
	13	9		4	1	
Funding panel member	Current			Previous		
	Chair	Deputy chair	Member	Chair	Deputy chair	Member
	5	2	7	1	1	5
Editor	Current			Previous		
	15			2		
University academic position held	Professor	Research Associate		Fellow	None	
	24	1		1	1	

Key: *CI* chief investigator, *CTU* clinical trials unit, *S* surgeon, previous, no longer practicing in a clinical role

**Fig. 2:**
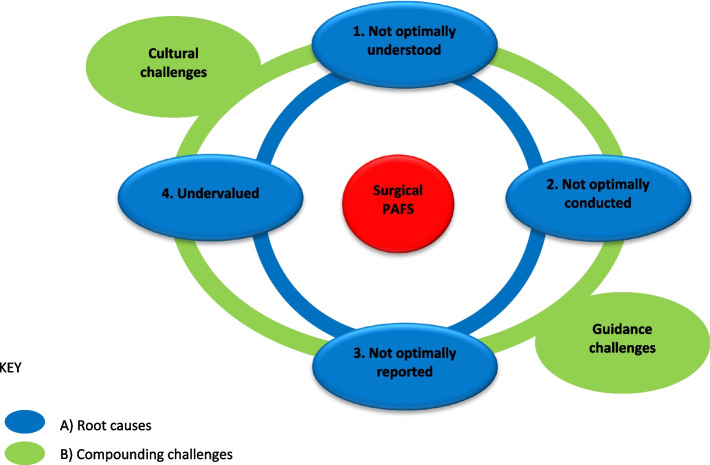
Cyclical model of sub-optimisation of PAFS illustrating the linked and co-influential root causes and compounding challenges underpinning why surgical PAFS are not currently optimised

As shown in Fig. 1 and described in the methods, phase 3 of this work synthesised the findings from our published 10-year review of NIHR-funded surgical PAFS [24], and the *n* = 27 qualitative interviews with key stakeholders, as well as available methodological guidance on PAFS [1, 8, 19, 20, 25–29]. This synthesis resulted in four root causes for why PAFS in surgery are  not currently optimised as shown in Fig. 2. These root causes emerged from the data as issues relating to (1) understanding the full scope of PAFS; (2) the design and conduct of PAFS; (3) the reporting of PAFS; and (4) the undervaluation of PAFS by surgeons, journal editors, academic institutions and sometimes funders. In addition to the root causes identified for why PAFS in surgery are not currently optimised, compounding factors were identified which are linked to both the root causes and to each other. These factors relate to the challenges of (1) current guidance and (2) cultural issues surrounding both surgical research in general and PAFS more specifically. These challenges can impact at different and multiple points in the cycle, and thereby act as barriers to improving research practice (see Fig. 2). Each of the root causes and compounding factors identified by this work are described in detail below, supported by participant quotes (see Table 2) and references to the other data sources synthesised to produce these results.

**Table 2:** Quotes from interview participants

	Quote number in text	Professional Role(s)	Quote
Root cause 1. Surgical PAFS are not optimally understood	1	Surgeon	I wouldn’t be a fan of doing a pilot study to see whether the design of the study worked…otherwise people are gonna go ‘oh what’, if you’ve not given them an answer; surgeons are pretty binary
	2	Methodologist/funder	My pet hate is something described as a pilot study. When you read it, all of the objectives and everything else reads as if it’s a definitive study but with a massive, thumping great effect size and actually it’s nothing of the sort. It means, we wanted to do the full study but we couldn’t afford it, or couldn’t find enough patients so we’ve done something, we’ve called it pilot, and yes it’s neither fish nor fowl
	3	CTU Director	The more uncertainty you’ve got, the more you need a pilot, and if you’re uncertain about surgeon equipoise, uncertain about patient equipoise, uncertain about the actual intervention then, compliance whatever, then that increases the probability you need a pilot
	4	Surgeon	Well it’s all about recruiting isn’t it, at the end of the day? And the trial’s got to be attractive to clinicians and the staff who are addressing the patients. So, anything that obstructs recruitment has to be addressed in a pilot study, I would have thought…
Root cause 2. Surgical PAFS are not optimally conducted	5	Surgeon/funder	I think the truth is most, many surgeons even those involved in trials don’t actually understand what feasibility and pilot work is and confuse it with a… it’s just a smaller trial, well no it’s not, it has a completely different role, maybe it’s stepping stone role but a very different role […] so, it’s not universal, but I think actually if you ask me what the average surgeon understood by that, they wouldn’t have a clue really…
	6	Methodologist/funder	I think the key list of things is also a bit of a stumbling block, and that’s in the NIHR feasibility definition, they have this list of things, and I think people think they’ve got to do that
	7	Funder	The area that’s probably more neglected, is the feasibility of the intervention and again there’s often a lack of appreciation of the complexity of interventions, lack of awareness of guidance in relation to evaluation of complex interventions, and often a naïve assumption that an intervention that’s been used by an expert in a specialist centre can simply be taken off the shelf and implemented throughout the NHS. And you really need to know about implementation before you start a full-scale pragmatic evaluation
Root cause 3. Surgical PAFS are not optimally reported	8	Surgeon/funder	With my journal editing hat on, I spend a lot of the time trying to persuade investigation groups so, they’ve actually done a pilot study and please report it as such. Even though they try and sell it to you as a definitive project with 20 patients in each group for a complex intervention
Root cause 4. Surgical PAFS are undervalued	9	CTU Director	I do think there are probably some people out there who spend their career doing pilots and don’t move forward into doing the definitive trial which is harder
	10	CTU Director	Sometimes you see pilot feasibility work, is seen as an end in itself […] [Funders have] funded masses of feasibility studies, most of which have gone nowhere and produced nothing of any interest […] I look at these things and say well, why would we go for these things? They’re three years of work, they’re usually very thin on resource, they’re a lot of hard work, and at the end of it we get nothing out of it that is of any use to us […] You can write up your feasibility work, it will go into the Ruritanian Journal of Unreproducible Results, nobody will ever read it again and it ticks a box for our masters that we’re generating income, but it doesn’t generate us any really top-class research output, so most of it goes nowhere
	11	CTU director/funder	Regrettably the university won’t see this as being an important paper because, nobody’s going to see it as being three or four star […] it ends up in a low key journal, they look at it, you haven’t collected any data, you haven’t got really hard outcomes other than saying it can’t be done or it can be done, we’re not interested, where’s the main data?
	12	CTU director/funder	There’s a lot of confusion about what people should be doing in these studies, and I think a lot of that comes from misunderstandings of journal editors and reviewers and trying to push people in a certain direction
	13	CTU director	All the science, all the clever stuff is in the protocol paper, which counts for nothing. And actually, in the feasibility work, because it’s all the positive feasibility work that got you to the point that you could do the main study. It’s where all the clever stuff is. The wonderfully concise paper in ‘The Lancet’ says, we tested it, we found a load of people with whatever it is they’ve got, we gave them whatever these two interventions were and either it worked, or it didn’t. I mean there’s nothing scientific about that
Compounding factor 1: guidance challenges	14	CTU director/funder	I think they [the definitions] push people into particular boxes, whereas different questions are better answered using different approaches. As for almost any research question you care to name there are pros and cons to different approaches but here, the question you’re answering is ‘what’s stopping me doing the main trial'
	15	Methodologist/funder	There are some nice papers actually on pilot and feasibility studies, particularly to do with sample size and I think they’re a bit… I mean they are very useful if you’re a methods person, I think they’re a bit tricky if you’re not […] so they’re good, but it’s obvious from the stuff we get from the [name of funder], that that message is not getting across through those articles in a way that is understandable
	16	Surgeon/funder	I struggle with that division between feasibility and pilot despite having read and re-read all the crap describing the differences, actually I don’t think the differences are particularly meaningful […] that division is probably not important but only exists, yes in funding scheme applications
	17	Methodologist/funder	I think there’s a bit of a failure on our part, meaning methods people like me, to translate for want of a better word, our stuff into a format that people who really have got better things to do, to use. They’ve got better things to do, than read through half a dozen papers. What they really want is to distil the key things that they really, really need, so they can build it into their idea
	18	Methodologist	In my opinion most surgeons haven’t got as far as pilot and feasibility studies, there wasn’t very much awareness of them […] I got the impression that not many people were using the IDEAL framework and weren’t aware of it […] They seem to be getting away with doing mini randomised controlled trials and, as I’ve described, they shouldn’t really be doing that […] I felt that they hadn’t got as far as doing pilot and feasibility studies, or embracing them in their work
	19	Surgeon/funder	I mean working within a trials unit to run these sorts of trials, you think is absolutely essential really, you can’t imagine working outside of it. I mean I can’t, but I know most, most clinicians do work outside and they have to. There’s a huge barrier between getting access to the trials units, getting CTUs to talk to you. Even well-established people are having trouble engaging with their CTU
	20	Surgeon	The major stumbling block is the fact that a lot of bodies require you to have a clinical trials unit, and the clinical trials units are often too expensive
Compounding factor 2: cultural challenges	21	Funder	Deciding whether a trial is worthwhile involves judging value for money and that value for money judgement has to be made from the point of view of the health service and the funder. There are one million-pound questions that are worth one million pounds, and there are five million-pound questions, or even ten million pound questions and we will look at the question, we will judge what it is. But we then need to know how much will a definitive trial cost. Because if a definitive trial is going to cost ten million pounds, and it’s only a one-million-pound question, then there’s no point funding the standalone pilot and, again, I think this is often underappreciated
	22	Surgeon	You’ll have one token surgeon with a grant giving body, who I should think, often, is not terribly diplomatic or very experienced in that sort of thing and it’s so competitive. Not just at the grant giving money for trials stage, but at the next level up where we’re going to spend our money, and translational studies and other types of sexy sounding personalised medicines and the humdrum randomised trial is hard to do. If they put their money into knock out mice, teams who know what they’re up to and have got to mould the work, crank that handle, they get the money and out comes a ‘Nature’ paper and we’re… it’s very hard for us to compete with that
	23	Surgeon	In terms of funding there are all sorts of funding streams that I’ve used in the past, including industry, and the major stumbling block is the disparity between what the funding bodies actually tell you, how they’re all interested in surgical research and how the minority of surgical research gets funded, and therefore, we’re all mobilising ourselves to make sure that that’s reversed. The fact that that’s not the case, and they’re completely disinterested in surgical research… I can say that safely across the board. I think it’s fair to say that a lot of charities are completely disinterested in anything that’s of clinical value, or that involves surgical research
	24	Surgeon/Funder	Speaking candidly, the big companies have got no interest in… in fact it’s almost a disincentive to do head to head comparisons of their technology against whatever other interventions are out there, and because there was no requirement for them to do so and they never actually developed… what you discover is they’ve got no infrastructure to do that
	25	Surgeon	The difference is that the pharmaceutical industry, not only has more funding, but has the requirement to carry out the work, whereas the device industry has got less money, but has plenty of money, but has no requirement to carry out the work. There’s a lack of a regulatory requirement
	26	Methodologist/Funder	You could get up to £300,000 for a feasibility study and after that you’re looking at NIHR money, so if you wanted £2 million, the only place you have to go is NIHR. HTA really, which means there’s a delay then of at least a year, and probably more than that. So, it really stretches out the development of that trial
	27	Surgeon	So if you do it in the linear way, the way you’re supposed to, we’d all be dead before you finished the main trial, which again comes back to my point about the present structure, is just too inefficient
	28	CTU Director	Things will move on, and it’s pointless to do a whole bunch of small pilot or feasibility studies, and then actually the question has moved on by the time we’ve worked out whether you can (laugh)…

Key: *PAFS* pilot and feasibility studies, *NIHR* National Institute for Health Research, *NHS* National Health Service, *CTU* clinical trials unit, *HTA* Health Technology Assessment

#### Root cause 1: surgical PAFS are not optimally understood

A key finding from all data sources was that the purpose and scope of PAFS were not well understood by the surgical research community. The analysis of NIHR-funded PAFS protocols [24], for example, demonstrated that nearly a quarter of PAFS studies planned to conduct formal hypothesis testing (8/35, 23%). Interview data confirmed that many surgeons perceived PAFS to be small underpowered RCTs, designed and reported with the opportunity to test certain outcomes including safety and effectiveness [see Table 2 Quotes 1 and 2]. Whilst methodologists understood the full scope of PAFS to explore the breadth of areas of uncertainty about a potential main trial [see Table 2 Quote 3], surgeons were generally less specific about the multiple areas of uncertainty that could be explored and tended to focus on recruitment as the primary area of uncertainty that could be addressed [see Table 2 Quote 4].

#### Root cause 2: surgical PAFS are not optimally conducted

Misunderstanding of what PAFS are and why they should be done impacts the range of areas of uncertainty that surgeons will seek to explore in PAFS. Consequently, PAFS in surgery are not optimally conducted [see Table 2 Quote 5]. In particular, there is a tendency for surgical PAFS [24] to focus on issues that are generic to all trials, such as recruitment, rather than exploring and addressing key uncertainties of specific relevance to surgical trials, such as intervention stability and the learning curve. Interviews with methodologists highlighted that surgeons applying for funding to undertake PAFS tended to follow ‘example’ lists provided by funders of what to consider investigating in PAFS, rather than thinking about the specific needs of their trial [see Table 2 Quote 6]. The importance of exploring the intervention protocol (which also includes careful consideration of co-interventions) in surgical PAFS was particularly underappreciated [see Table 1 Quote 7].

#### Root cause 3: surgical PAFS are not optimally reported

Both the quantitative and qualitative work provided evidence that surgical PAFS are currently not well reported. Data from the review of NIHR-funded surgical PAFS illustrated that PAFS in surgery is under-reported, with only two-thirds of surgical PAFS studies funded by the NIHR between 2005 and 2015 publishing study findings [24]. In addition, interview findings demonstrated that PAFS are still masqueraded as full RCTs when submitted to journals and that underpowered RCTs are badged as PAFS *a posteriori *[see Table 2 Quote 8].

#### Root cause 4: surgical PAFS are undervalued

The interview study provided an explanation for why PAFS might be sub-optimally conducted and reported [24], by illustrating that PAFS were undervalued by all key stakeholder groups. Funders, for example, perceived that many PAFS had historically been conducted as standalone pieces of work with no intention of the study team or funders to progress to a main trial. PAFS were consequently undervalued as being ineffectual and not worth investment [see Table 2 Quotes 9 and 10]. Similarly, academic institutions were perceived to undervalue PAFS, considering them low-impact studies, which do not contribute significantly to the Research Excellence Framework (REF) as high-impact papers, and often have no outputs at all [see Table 2 Quote 11]. This is perpetuated by journal editors and peer reviewers, some of whom undervalue PAFS and consider them of limited interest as they do not offer definitive practice-changing results [see Table 2 Quote 12]. Such editorial practice may perpetuate the cycle of misunderstanding; if definitive results are requested, authors may feel compelled to produce them to achieve publication, thus small underpowered RCTs veiled as PAFS will continue to litter the literature, which further perpetuates misunderstanding [see Table 2 Quote 13]. Whilst there is now this journal dedicated to the reporting and publication of PAFS (The Journal of Pilot and Feasibility Studies [32]), without investment from journal editors, academic institutions and funders to drive the importance of accurately publishing pre-trial work, the cycle of sub-optimisation of PAFS will continue.

#### Compounding factor 1: challenges with current guidance

The interviews identified challenges with both guidance provided by funders, and information in the methodological literature conceptualising the types and purposes of PAFS and describing methods for reporting PAFS [8, 25–29]. The funder guidance was perceived as being limited, variable and sometimes contradictory to the definitions given in the methodological guidance [see Table 2 Quote 14]. Whilst most methodologists recognised the extensive methodological work already undertaken [8, 25–29], many felt this work to be inaccessible and poorly disseminated to surgeons [see Table 2 Quote 15].

There was limited awareness of the existence of the current methodological work amongst surgeons. The few surgeons who did mention this perceived the methodological literature as largely theoretical, generic and difficult to operationalise, thereby making it mostly unhelpful [see Table 2 Quotes 16]. This finding indicates that the methodological work is poorly understood and not widely acknowledged beyond the methodological community [see Table 2 Quote 17]. Guidance from the IDEAL collaboration [19, 33], widely considered as the conceptual work most aligned with surgeons and surgical trials, was not perceived in the interview study to be widely accepted or utilised amongst surgeons [see Table 2 Quote 18]. It is encouraging that the newest NIHR guidance first published in 2019 [34] does now signpost and reference the underpinning methodological work in this area.

If available methodological guidance is not effectively operationalised to be of practical use to surgeons [see Table 2 Quote 17], its inaccessibility is compounded, further adding to confusion so that it is consequently misunderstood or ignored. In addition, surgeons recognised significant barriers to being able to access Clinical Trials Units (CTUs) and methodological expertise, which was often perceived as not possible within PAFS funding envelopes [see Table 2 Quotes 19 and 20].

#### Compounding factor 2: cultural challenges

Many of the existing challenges for surgical trials have already reported [2–7] impact on PAFS and, in doing so, make PAFS potentially even more relevant in surgery. There has been a blossoming culture of surgical research partnerships and cross-specialty collaboration in recent years through, for example, the formation of nationwide surgical trainee research collaboratives and the RCS of England Surgical Trials Initiative [35]. Such cultural changes have undoubtedly contributed significantly to raising the profile of surgical research and, more specifically collaboratively conducting surgical trials [36–38]. However, of all clinical research funded by the NIHR Health Technology Assessment (HTA) and Research for Patient Benefit (RfPB) programmes from 2005 to 2015, only 10.4% (140/1341) were studies where surgery was the main intervention [24]. Most funders felt that the reason surgical research was less frequently funded was because the questions being asked were not important enough to the National Health Service (NHS) or to patients [see Table 2 Quote 21]. In contrast, surgeons perceived a lack of surgical representation on funding panels, and competition with translational science and experienced research teams for funding, as significant barriers to fair funding opportunities [see Table 2 Quotes 22 and 23].

Surgeons perceived other barriers to funding related to the relative lack of regulation for the formal evaluation of new surgical procedures and surgical devices. This was observed to have led to a lack of research infrastructure within the industry, resulting in fewer avenues for funding surgical research when compared, for example, to pharmaceutical research [see Table 2 Quotes 24 and 25].

Most funders still offer ‘uncoupled’ funding, where a PAFS is funded without a firm promise of funding for a subsequent main trial. This system was perceived by most interview participants as inefficient, due to the additional time and resources needed to perform standalone pre-trial work, and therefore a further barrier to completing PAFS [see Table 2 Quote 26].

Finally, both surgeons and methodologists perceived undertaking PAFS to lengthen the process of trial research, meaning answers to important questions took longer to attain. Consequently, it was considered that the research question may become obsolete before pre-trial work is completed, particularly in fast-moving clinical areas such as surgery [see Table 2 Quote 27 and 28].

## Discussion

This is the first published work to specifically consider current research practice for PAFS in surgery and to explore the explicit challenges and barriers preventing optimal conduct of PAFS in surgery. This work has identified four key areas for improvement to research practice necessary to optimise future PAFS in surgery. These have informed the development of broad recommendations, summarised in Table 3, which require a wider cultural shift in research practice amongst funders, academic institutions, regulatory bodies and journal editors, as well as amongst surgeons. Whilst the recommendations from this work are focused on PAFS in surgery, many may be relevant to the wider context of complex interventions as a whole. As part of a future consensus process, this question could perhaps be addressed, especially in light of similar work in other areas [39–41].

**Table 3:** Recommendations for change to improve wider research practice around P/FS

	Recommendation	Further detail	Issues to consider
Education	Improved guidance on designing and conducting PAFS	Multi-disciplinary team-led, consensus-based guidance endorsed by funders, regulatory bodies and journals	How to operationalise theoretical/conceptual guidance specifically for application by clinicians in practice
	Grassroots training for surgeons from earlier in their career	Training in trials methodology through courses, conferences, publication and guidelines	How to ensure effective collaboration between clinicians and methodologists
Collaboration	Collaboration of surgeons with methodologists and CTUs	Working closely with methodologists and CTUs from earlier in the research process to ensure the future main trial is in sight	Practicalities of funding collaborations How to optimise PPI when designing and conducting PAFS for surgical trials
	Accessibility of CTUs and methodology support	Highlight where to go/who to ask for assistance in each geographical area in the new guidance	Consider what level of method support is enough for PAFS
Funding	Improved efficiency of funding structure	More joined-up funding so no lag time between successful PAFS and main trial	Consider more programmes offering staged funding like NIHR PGfAR to improve efficiency and reduce waste How to associate decision-making between local RfPB committees and national funders of definitive trials
	Raising the profile of the importance of funding surgical studies	To achieve proportional funding More surgeons on funding panels Regulatory requirements for industry to contribute to surgical research	How to promote surgical involvement on funding panels
Dissemination	Funder requirement to publish PAFS	Publication in journals and/or through publicly available funder reports	How to fund process of publication
	Journal editors stop publishing underpowered RCTs as PAFS or PAFS as underpowered RCTs	Both wrong. Educate through guidance	Consider involving editors of surgical journals in the process for producing guidance
	Academic institutions to value PAFS as potentially essential for main trial development	PAFS may not be 3 or 4* REF rated alone, but should be recognised for the often pivotal role they play in the success of the definitive trial. If academic institutions do not value PAFS, researchers will not value disseminating their findings	How to engage academic institutions in considering the value of PAFS

Key: *PAFS* pilot and feasibility studies, *CTUs* clinical trials units, *PPI* patient and public involvement, *NIHR* National Institute for Health and Care Research, *PGfAR* Programme Grants for Applied Research, *RfPB* Research for Patient Benefit, *RCTs* randomised controlled trials, *REF* Research Excellence Framework

Examining the literature, it seems PAFS may be less commonly done in countries outside the UK. A systematic review by the methodology group which produced the conceptual framework of the definitions of PAFS, looked at the quality of reporting of 18 pilot and feasibility cluster randomised trials conducted and published between 2011 and 2014 [42]. This study found that half (56%) were set in the UK, with all other countries represented only once, apart from Canada (three studies) and the United States of America (USA) (two studies). In addition, it was noted in our study, that the UK-produced methodological guidance [8, 26] was perceived to have not been incorporated into practice by authors from overseas yet.

Whilst the focus of this work was entirely on research and funding practice in the UK, it is perhaps reasonable to suggest that the UK is leading the way in developing a methodology for the design and conduct of PAFS and that with further exploration and collaboration, the findings of this research could well be relevant to researchers in other countries. A limitation of this work is that only senior surgeons and methodologists were sampled. Surgeons leading the trainee surgical research collaboratives and research-naïve surgeons for example may have provided differing perspectives and potential solutions to the challenges encountered. However, the well-documented issues with inappropriate reporting of both underpowered RCTs as PAFS and vice versa [43–45] indicate a widespread misunderstanding of the value and purpose of PAFS. It was therefore deemed important to concentrate on extracting data from the most experienced and data-rich sources, hence focusing on experienced participants for the interviews, and systematic analysis of NIHR-funded PAFS (as opposed to performing a traditional systematic or narrative review of the literature). In addition, whilst patients were not involved in this work as the aim was to specifically explore the methodological and cultural barriers and challenges of completing PAFS from a professional perspective, involving patients in the design and delivery of PAFS is vitally important, and future work in the area will need to include the patient perspective.

## Conclusion

**Fig. 3:**
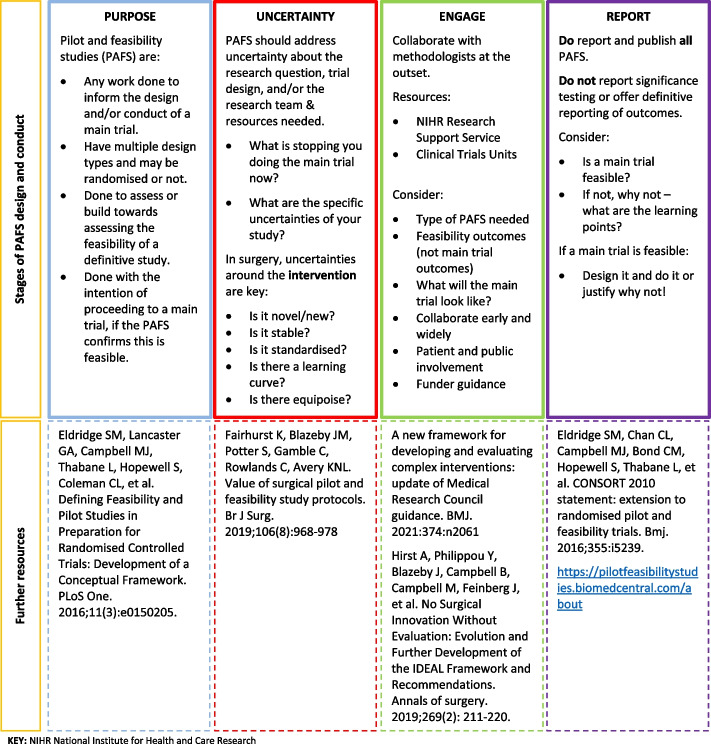
Top Tips for surgeons designing and conducting pilot and feasibility studies

This work identified the need for accessible, operationalised guidance for surgeons designing and conducting surgical PAFS. Our ‘Top Tips’ guidance tool for surgeons (Fig. 3) offers a practical framework for surgeons designing and writing funding applications for PAFS. The guidance operationalises and bridges the current gap between the available methodological guidance and the broader recommendations for improving research practice made here (see Table 3). These top tips include defining the purpose of PAFS, identifying uncertainties of specific relevance to surgery to be considered and engaging with methodologist support early and systematic reporting of PAFS, with references to key methodological resources. Both the recommendations for cultural changes (Table 3) and the practical guidance tips for surgeons (Fig. 3) are intended to optimise future best research practice around the design and conduct of surgical PAFS. Adoption of these recommendations will, therefore, facilitate successful and efficient surgical trials in the future and, ultimately, improve the evidence base for surgeons and patients.

## Supplementary Information

**Additional file 1. Appendix 1.** Semi-structured interview topic guide.

## Data Availability

The datasets generated and analysed during the current study are stored under the provisions of the Data Protection Act 2018 and the University of Bristol requirements but are not publicly available due to the potentially sensitive nature of the data, and the risk of re-identification of participants. Data may be made available to bona fida researchers only, on reasonable request to the corresponding author after their host institution has signed a Data Access Agreement.

## Abbreviations

PAFS: Pilot and feasibility studies
RCTs: Randomised Controlled Trials
UK: United Kingdom
NIHR: National Institute for Health and Care Research
MRC: Medical Research Council
RCS: Royal College of Surgeons
CRUK: Cancer Research UK
CSO: Chief Scientist Office
ARUK: Arthritis Research UK
CTUs: Clinical Trial Units
IDEAL collaboration: Idea, Development, Evaluation, Assessment, Long-term follow-up
HTA: Health Technology Assessment
RfPB: Research for Patient Benefit
USA: United States of America
REF: Research Excellence Framework

## References

[1] Medical Research Council (MRC). Developing and evaluating complex interventions: new guidance. London: Medical Research Council; 2008.

[2] Ergina PL, Cook JA, Blazeby JM, Boutron I, Clavien PA, Reeves BC, et al. Challenges in evaluating surgical innovation. Lancet. 2009;374(9695):1097–104. 19782875 10.1016/S0140-6736(09)61086-2 PMC2855679

[3] McCulloch P, Taylor I, Sasako M, Lovett B, Griffin D. Randomised trials in surgery: problems and possible solutions. BMJ. 2002;324(7351):1448–51. 12065273 10.1136/bmj.324.7351.1448 PMC1123389

[4] National Cancer Research Institute (NCRI). Challenges and opportunities in surgical cancer research in the UK. London: National Cancer Research Institute; 2012.

[5] Cook JA. The challenges faced in the design, conduct and analysis of surgical randomised controlled trials. Trials. 2009;10:9. 19200379 10.1186/1745-6215-10-9 PMC2654883

[6] Byrne BE, Rooshenas L, Lambert HS, Blazeby JM. A mixed methods case study investigating how randomised controlled trials (RCTs) are reported, understood and interpreted in practice. BMC Med Res Methodol. 2020;20(1):112. 32398100 10.1186/s12874-020-01009-8 PMC7216481

[7] Farrokhyar F, Karanicolas PJ, Thoma A, Simunovic M, Bhandari M, Devereaux PJ, et al. Randomized controlled trials of surgical interventions. Ann Surg. 2010;251(3):409–16. 20142732 10.1097/SLA.0b013e3181cf863d

[8] Eldridge SM, Lancaster GA, Campbell MJ, Thabane L, Hopewell S, Coleman CL, et al. Defining feasibility and pilot studies in preparation for randomised controlled trials: development of a conceptual framework. Plos One. 2016;11(3):e0150205. 26978655 10.1371/journal.pone.0150205 PMC4792418

[9] Lancaster GA, Dodd S, Williamson PR. Design and analysis of pilot studies: recommendations for good practice. J Eval Clin Pract. 2004;10(2):307–12. 15189396 10.1111/j..2002.384.doc.x

[10] O’Cathain A, Thomas KJ, Drabble SJ, Rudolph A, Goode J, Hewison J. Maximising the value of combining qualitative research and randomised controlled trials in health research: the QUAlitative Research in Trials (QUART) study–a mixed methods study. Health Technol Asses. 2014;18(38):1–197 (**v-vi**). 10.3310/hta18380 PMC4781055 24914457

[11] National Institute for Health and Care Research (NIHR). Clinical Trials Toolkit. 2023. [Available from: https://www.ct-toolkit.ac.uk/routemap/feasibility-and-investigator-selection/]. Accessed 22 Aug 23.

[12] Al-Shahi Salman R, Beller E, Kagan J, Hemminki E, Phillips RS, Savulescu J, et al. Increasing value and reducing waste in biomedical research regulation and management. Lancet. 2014;383(9912):176–85. 24411646 10.1016/S0140-6736(13)62297-7 PMC3952153

[13] Chalmers I, Bracken MB, Djulbegovic B, Garattini S, Grant J, Gulmezoglu AM, et al. How to increase value and reduce waste when research priorities are set. Lancet. 2014;383(9912):156–65. 24411644 10.1016/S0140-6736(13)62229-1

[14] Chan AW, Song F, Vickers A, Jefferson T, Dickersin K, Gotzsche PC, et al. Increasing value and reducing waste: addressing inaccessible research. Lancet. 2014;383(9913):257–66. 24411650 10.1016/S0140-6736(13)62296-5 PMC4533904

[15] Glasziou P, Altman DG, Bossuyt P, Boutron I, Clarke M, Julious S, et al. Reducing waste from incomplete or unusable reports of biomedical research. Lancet. 2014;383(9913):267–76. 24411647 10.1016/S0140-6736(13)62228-X

[16] Ioannidis JP, Greenland S, Hlatky MA, Khoury MJ, Macleod MR, Moher D, et al. Increasing value and reducing waste in research design, conduct, and analysis. Lancet. 2014;383(9912):166–75. 24411645 10.1016/S0140-6736(13)62227-8 PMC4697939

[17] Leon AC, Davis LL, Kraemer HC. The role and interpretation of pilot studies in clinical research. J Psychiatr Res. 2011;45(5):626–9. 21035130 10.1016/j.jpsychires.2010.10.008 PMC3081994

[18] Skivington K, Matthews L, Simpson SA, Craig P, Baird J, Blazeby JM, et al. Framework for the development and evaluation of complex interventions: gap analysis, workshop and consultation-informed update. Health Technol Assess. 2021;25(57):1–132. 34590577 10.3310/hta25570 PMC7614019

[19] McCulloch P, Altman DG, Campbell WB, Flum DR, Glasziou P, Marshall JC, et al. No surgical innovation without evaluation: the IDEAL recommendations. Lancet. 2009;374(9695):1105–12. 19782876 10.1016/S0140-6736(09)61116-8

[20] Hirst A, Philippou Y, Blazeby J, Campbell B, Campbell M, Feinberg J, et al. No surgical innovation without evaluation: evolution and further development of the IDEAL framework and recommendations. Ann Surg. 2019;269:211–20. 29697448 10.1097/SLA.0000000000002794

[21] Fairhurst KAK, O’Connell Francischetto E, Metcalfe C, Blazeby J. How can pilot work optimally inform surgical RCTs? A review of current evidence. Trials. 2015;16(Suppl 2):17.25622970

[22] O’Connell Francischetto EAK, Metcalfe C, Williamson P, Gamble C, Blazeby J. Optimising the design and evaluation of pilot work to inform the main trial: a review of current evidence and consideration of future practices. Trials. 2013;14(Suppl 1):O17.

[23] McCulloch P, Feinberg J, Philippou Y, Kolias A, Kehoe S, Lancaster G, et al. Progress in clinical research in surgery and IDEAL. Lancet. 2018;392(10141):88–94. 29361334 10.1016/S0140-6736(18)30102-8

[24] Fairhurst K, Blazeby JM, Potter S, Gamble C, Rowlands C, Avery KNL. Value of surgical pilot and feasibility study protocols. Br J Surg. 2019;106(8):968–78. 31074503 10.1002/bjs.11167 PMC6618315

[25] Eldridge SBC, Campbell M, Lancaster G, Thabane L, Hopewell S. Definition and reporting of pilot and feasibility studies. Trials. 2013;14(Supplement 1):O18.

[26] Eldridge SM, Chan CL, Campbell MJ, Bond CM, Hopewell S, Thabane L, et al. CONSORT 2010 statement: extension to randomised pilot and feasibility trials. BMJ. 2016;355:i5239. 27777223 10.1136/bmj.i5239 PMC5076380

[27] Eldridge SM, Chan CL, Campbell MJ, Bond CM, Hopewell S, Thabane L, et al. CONSORT 2010 statement: extension to randomised pilot and feasibility trials. Pilot Feasibil Stud. 2016;2:64. 10.1186/s40814-016-0105-8 PMC5154046 27965879

[28] Eldridge SBC, Campbell M, Hopewell S, Thabane L, Lancaster G, Coleman C. Defining feasibility and pilot studies in preparation for randomised controlled trials: using consensus methods and validation to develop a conceptual framework. Trials. 2015;16(Suppl 2):087. 10.1371/journal.pone.0150205 PMC4792418 26978655

[29] Thabane L, Hopewell S, Lancaster GA, Bond CM, Coleman CL, Campbell MJ, et al. Methods and processes for development of a CONSORT extension for reporting pilot randomized controlled trials. Pilot Feasibil Stud. 2016;2(1):1–13. 10.1186/s40814-016-0065-z PMC5153862 27965844

[30] Braun VCV. Using thematic analysis in psychology. Qual Res Psychol. 2006;3:77–101.

[31] http://www.qsrinternational.com/nvivo [Accessed 22.08.2023]

[32] Lancaster GA. Pilot and feasibility studies come of age! Pilot Feasibil Stud. 2015;1(1):1–4. 10.1186/2055-5784-1-1 PMC5842886 29611687

[33] IDEAL Collaboration. http://www.ideal-collaboration.net/ [Accessed 09.11.2022].

[34] National Institute for Health and Care Research (NIHR). RfPB programme guidance on applying for feasibility studies [Available from: https://www.nihr.ac.uk/documents/nihr-research-for-patient-benefit-rfpb-programme-guidance-on-applying-for-feasibility-studies/20474?pr]. Accessed 2 Sept 2019.

[35] Royal College of Surgeons of England (RCSEng). https://www.rcseng.ac.uk/standards-and-research/research/surgical-trials-initiative/. Accessed 28 June 2017.

[36] Royal College of Surgeons of England (RCSEng). Surgical Research Report 2017–2018. 2017 [Available from: https://www.rcseng.ac.uk/library-and-publications/rcs-publications/docs/rcs-surgical-research-report-2017/]. Accessed 9 Nov 2022.

[37] Pinkney TD, Calvert M, Bartlett DC, Gheorghe A, Redman V, Dowswell G, et al. Impact of wound edge protection devices on surgical site infection after laparotomy: multicentre randomised controlled trial (ROSSINI Trial). BMJ. 2013;347:f4305. 23903454 10.1136/bmj.f4305 PMC3805488

[38] Jamjoom AA, Phan PN, Hutchinson PJ, Kolias AG. Surgical trainee research collaboratives in the UK: an observational study of research activity and publication productivity. BMJ Open. 2016;6(2):e010374. 26846898 10.1136/bmjopen-2015-010374 PMC4746473

[39] Peter Craig AM, Browne S, Simpson SA, Wight D, Robling M, Moore G, Hallingberg B, Segrott J, Turley R, Murphy S, Moore L. Development of guidance for feasibility studies to decide whether and how to proceed to full-scale evaluation of complex public health interventions: a systematic review. Lancet. 2018;392:7. 29854417 10.1186/s40814-018-0290-8 PMC5971430

[40] Moore L, Hallingberg B, Wight D, Turley R, Segrott J, Craig P, et al. Exploratory studies to inform full-scale evaluations of complex public health interventions: the need for guidance. J Epidemiol Community Health. 2018;72(10):865–6. 30030296 10.1136/jech-2017-210414 PMC6161652

[41] Hallingberg B, Turley R, Segrott J, Wight D, Craig P, Moore L, et al. Exploratory studies to decide whether and how to proceed with full-scale evaluations of public health interventions: a systematic review of guidance. Pilot Feasibil Stud. 2018;4:104. 10.1186/s40814-018-0290-8 PMC5971430 29854417

[42] Chan CL, Leyrat C, Eldridge SM. Quality of reporting of pilot and feasibility cluster randomised trials: a systematic review. BMJ Open. 2017;7(11):e016970. 29122791 10.1136/bmjopen-2017-016970 PMC5695336

[43] Arain M, Campbell MJ, Cooper CL, Lancaster GA. What is a pilot or feasibility study? A review of current practice and editorial policy. BMC Med Res Methodol. 2010;10:67. 20637084 10.1186/1471-2288-10-67 PMC2912920

[44] Loscalzo J. Pilot trials in clinical research: of what value are they? Circulation. 2009;119(13):1694–6. 19349331 10.1161/CIRCULATIONAHA.109.861625

[45] Shanyinde M, Pickering RM, Weatherall M. Questions asked and answered in pilot and feasibility randomized controlled trials. BMC Med Res Methodol. 2011;11:117. 21846349 10.1186/1471-2288-11-117 PMC3170294

