# Supplementary material for: Recommendations for optimising pilot and feasibility work in surgery
Source: Pilot Feasibility Stud. 2024 Apr 18;10:64. doi: 10.1186/s40814-024-01489-1 (PMC11025276; doi:10.1186/s40814-024-01489-1)
Supplement: Supplementary file 1 — Additional file 1. Appendix 1. Semi-structured interview topic guide. [file 40814_2024_1489_MOESM1_ESM.docx]

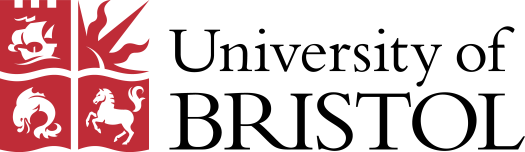

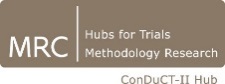
Kj

Participant ID Number:

**Appendix 1: Semi structured interview topic guide**

**Exploring Perceptions and Experiences of Pilot work for Surgical Trials: A Qualitative Research Study (PEPSTAR)**

**1. Introduction 2. General Information**

Your name & role Current & previous roles

Purpose and aims of the study e.g. surgeon, university, editor, funder, REC, trial team etc

Test audio recorder Specific areas of research interest

Explain will record interview

Written consent

**3. Topic areas** (Introduce each area as necessary)

A) General B) Importance

What is (you/in general) PW/FW? How important is PW?

What is (you/in general) IP/EP/NRFW? Why is it important?

When would you do internal/external pilot? Any examples of how/why important

What role does it have? How impacts on design/conduct/funding

What should its role be?

What issues should PW/FW consider?

Generic? (E.g. recruitment, retention, eligibility, protocol adherence, outcome assessment – selection/data collection & quality)

Specific to surgery? (E.g. developing innovation, standardisation, leaning curves, quality assurance, co–interventions, preferences/acceptability, blinding)

Progression criteria?

What/how decided/always met?/challenges

C) Experience D) Challenges/difficulties

Examples of specific studies What challenges in general with PW/FW?

Why i.e. purpose/rationale *Why* are they challenges?

Objectives at start – did they change? Examples of specific challenges/difficulties

Why internal/external/both? What were these? Methodological/cultural?

Participant’s role Why did they occur?

Design How dealt with?

How design decided? By who? Learning points?

Did design change? Do anything differently?

How? Why? Any remaining uncertainties about a main trial?

Conduct

Discussions with funders E) Essential components

When? Why? Outcome? Any key components to the design? (Cost effectiveness?)

Any key processes for conduct?

Who needs to be involved?

**4. Closing the interview**

Thank them for their time - Any further questions/comments?

Re-iterate may be in touch about inviting them to take part in a consensus process

Example questions:

1. **General**

- What do you/other people think PW/FW is?
- Can you expand on the meanings of the terms IP/EP and NRFW?
- How would you choose when to do an internal or external pilot?
- What role should PW/FW have in the context of trial design?
- What role does PW/FW have currently?
- What are the reasons for doing PW/FW?

OR What is important for PW/FW to show before you go ahead with a MT?

OR What sort of questions (about whether a MT is possible) should PW/FW be answering?

- Where does your understanding of PAFS come from?
- Are you aware of any guidance on the design and conduct of PAFS?
  - What guidance would you find helpful? Who should create/endorse that guidance?
- Do you have any experience of determining progression criteria for PW?
  - What do you think these should be? What are their purpose?
  - How should they be decided? How are they decided in your experience?
  - Do you have examples where an IP has progressed to a MT with PC being met/not met?
  - Do you think there are any specific challenges around selecting and using PC for IP studies?

1. **Importance & Impact**

- Do you think it is important to always do PW/FW before a MT?
  - Why do you think it is important? Examples of importance is from your own/others work?
  - When might PW/FW might not be necessary? Examples?
  - Do you think a PW/FW showing a MT is not viable is valuable?
- How does PW/FW impact on MT design/conduct/funding?
- Should PAFS be published? Why?

1. **Experience**

- Could we talk about some of your own experiences of designing/conducting/funding PW/FW?
  - What was the rationale for doing X study?
  - Why did you choose to do an internal/external pilot?
- How was the design of X study decided?
  - Who was involved in the design?
  - Did the design change during the study? Why?
- Could you describe any positive or negative experiences you had with funding bodies in the context of pilot/feasibility studies?
  - Any difficulties getting funding for X study?
  - Why do you think there were difficulties?

1. **Challenges**

- We have discussed some of these already, but could you summarise in your opinion, what you think the main challenges of doing (designing/conducting) PW/FW are?
  - Methodological/cultural?
  - Why do you think there are these challenges?
  - How did you/the trial team deal with these challenges?
  - Would you do anything differently if you were planning the PW/FW study again?
- Were there any remaining uncertainties about the MT once the PW/FW was completed?

1. **Essential components**

- We have talked about some important components to PW/FW already, but could you summarise/comment on what in your opinion, you think the key components to designing successful pilot/feasibility studies are?
- Do you think it is important to consider cost effectiveness in PW/FW?
- Do you think there are any key processes/personnel for the conduct of PW/FW?
